# Knowledge, Attitude, and Screening of Kidney Disease Among Asymptomatic Healthcare Students at a Tertiary Healthcare Center in Coastal Andhra Pradesh

**DOI:** 10.7759/cureus.90299

**Published:** 2025-08-17

**Authors:** Nishal A Kumar, VS Bhargav Pradeep Konakanchi, Advaita Veliyambra, Sumanth Gundraju, Srija Kadari, Vinod Kumar Reddy Murukuti, Sai Maneesha Thondur, Abhishek Kande

**Affiliations:** 1 General Internal Medicine, Andhra Medical College, Visakhapatnam, IND; 2 Internal Medicine, Andhra Medical College, Visakhapatnam, IND; 3 Emergency Medicine, Midland Metropolitan University Hospital, Birmingham, GBR; 4 Medicine, Manipal Hospitals, Bangalore, IND; 5 Medicine, Rangaraya Medical College, Kakinada, IND; 6 Internal Medicine, Malla Reddy Institute of Medical Sciences (MRIMS), Hyderabad, IND; 7 Internal Medicine, Anam Chenchu Subba Reddy (ACSR) Government Medical College, Nellore, IND; 8 Medicine, Anam Chenchu Subba Reddy (ACSR) Government Medical College, Nellore, IND

**Keywords:** chronic kidney disease (ckd), ckdu, healthcare students, knowledge-attitude-practice, proteinuria, screening, urine dipstick

## Abstract

Background

Chronic kidney disease (CKD) often begins silently and is typically identified only in its advanced stages. In regions such as North Coastal Andhra Pradesh, a high prevalence of CKD of unknown etiology (CKDu) has been reported. Early detection in asymptomatic individuals, especially among future healthcare professionals, is vital for prevention and timely intervention.

Methods

An observational study was conducted among 240 healthcare students, including medical and paramedical undergraduates. The participants were evaluated for knowledge, attitudes, and symptoms related to kidney disease. Urine dipstick testing was performed to screen for proteinuria and glycosuria. Data were analyzed using SPSS version 24 (IBM Corp., Armonk, NY).

Results

Among medical students, 6.1% had good knowledge, 81.6% had fair knowledge, and 12.2% had poor knowledge of kidney disease. In contrast, 58.3% of paramedical students had poor knowledge. Proteinuria was detected in 11.3% of male and 21.4% of female medical students; 8.3% of paramedical students also tested positive. Attitude toward kidney health, treatment preference, and organ donation varied significantly between groups.

Conclusion

A notable proportion of asymptomatic young healthcare students showed early urinary abnormalities. Knowledge gaps and varying attitudes emphasize the need for targeted education and routine screening initiatives in medical training.

## Introduction

The development of kidney disease often begins early in life, primarily triggered by modifiable risk factors [1]. The reported prevalence of chronic kidney disease (CKD) varies globally, with estimates ranging from less than 1% to 13% [2]. The etiology of CKD differs across various regions of India. Notably, certain areas within Andhra Pradesh, Odisha, and Goa report a high prevalence of CKD of unknown etiology (CKDu), a chronic interstitial nephropathy characterized by insidious onset and slow progression [3-5].

In particular, a high burden of CKDu has been documented in the coastal districts of Andhra Pradesh [6]. One such region is Uddanam in Srikakulam district, a fertile, subtropical low-altitude area in Southern Andhra Pradesh. Studies have raised concerns about the elevated incidence of CKD in this population, where major conventional risk factors, including diabetes, long-standing hypertension, and significant proteinuria, were absent in approximately 73% of affected individuals [7]. Early screening to identify risk factors, coupled with educational interventions aimed at promoting kidney health, represents a crucial opportunity to prevent or mitigate the progression of kidney disease before it reaches advanced stages [8-10]. Despite this, there are limited data on the implementation and impact of combined screening and educational programs, especially among younger, potentially vulnerable populations such as healthcare students.

This study aims to evaluate the level of knowledge, attitudes, and screening outcomes for kidney disease among asymptomatic medical and paramedical undergraduate students; to identify early urinary abnormalities through urine dipstick testing; and to promote broader awareness and understanding of various aspects of kidney disease through this combined educational and screening initiative.

## Materials and methods

This observational study was conducted over a period of two months at Andhra Medical College, Visakhapatnam. The Institutional Ethics Committee of Andhra Medical College issued approval 200/IEC AMC/JUNE 2021. A total of 240 students aged 18-24 were enrolled in the study using a purposive sampling method. The sample included medical and paramedical students from various academic streams who met the inclusion criteria and consented to participate during the study period. The inclusion criteria are as follows: all asymptomatic, i.e., the absence of a prior diagnosis or clinical presentation of known kidney disease, undergraduate medical and paramedical students who were willing to participate and provided informed consent during the study period. The exclusion criteria are as follows: students with a known diagnosis of any form of kidney disease or currently undergoing treatment for kidney-related disorders. The participants were provided with a brief introduction outlining the importance and purpose of the study. Data collection was carried out using a structured information sheet that recorded basic demographic details, kidney disease symptom profile, personal and family history of kidney disease, treatment history, body weight, and blood pressure (BP). Blood pressure was measured using a standardized manual sphygmomanometer with the participant seated and at rest. Two readings were taken at a five-minute interval, and the average was recorded for analysis. A systolic BP of ≥140 mmHg or diastolic BP of ≥90 mmHg was considered abnormal, in accordance with Joint National Committee (JNC) 7 guidelines. Additionally, a pre-validated questionnaire was administered to assess participants' knowledge, attitudes, and perceptions regarding kidney disease.

For screening, freshly collected midstream spot urine samples were collected and analyzed using the Uristix method to detect the presence of protein and glucose. In cases where proteinuria was detected, further evaluation was performed through urine sediment examination. The participants with abnormal findings were referred to the department of urology for further management. Qualitative data were expressed as frequencies and percentages. Comparative analyses were conducted using the chi-square test where applicable. All statistical analyses were performed using the SPSS software, version 24 (IBM Corp., Armonk, NY). 

## Results

A total of 240 participants were assessed for their knowledge, general attitudes, and screening for kidney disease. Among these, 180 were medical undergraduate students, and 60 were paramedical students. The study group included 110 male and 130 female participants. All participants underwent screening for symptoms suggestive of chronic kidney disease and urine dipstick testing.

Knowledge of kidney disease

Among the 180 medical undergraduate students, 6.1% (n = 11) demonstrated good knowledge of kidney disease, while 12.2% exhibited poor knowledge. The majority, 81.6%, had a fair level of knowledge on the subject. Among the 60 paramedical students, only 1.6% had good knowledge, while 40% demonstrated fair knowledge. A majority (58.3%) of paramedical students had poor knowledge regarding kidney disease.

Urine dipstick screening

While 240 students were enrolled, nine participants were unable to provide urine samples on the day of screening due to reasons such as menstruation, absence, or unwillingness. As a result, urine dipstick testing was completed for 231 participants. A total of 231 participants underwent urine dipstick screening, comprising 171 medical students and 60 paramedical students. The group included 110 men and 121 women. Among male medical undergraduate students (n = 106), glucose was detected (traces) in 2.9% (n = 3), and proteinuria (traces) was observed in 11.3% (n = 12). Among female medical undergraduate students (n = 65), glucose was detected as traces in 1.5% (n = 1) and 1+ in 1.5% (n = 1). Proteinuria was observed as traces in 18.4% (n = 12), 1+ in 1.5% (n = 1), and 3+ in 1.5% (n = 1). Among paramedical students (n = 60), 8.3% (n = 5) showed proteinuria (traces), while no cases of glycosuria were detected (Table 1).

**Table 1:** Combined Urine Dipstick Test Results for Glucose and Protein

Group	Glucose (Negative)	Glucose (Trace)	Glucose 1+	Glucose 2+	Glucose 3+	Protein (Negative)	Protein (Trace)	Protein 1+	Protein 2+	Protein 3+
Male medical students (n = 106)	103	3	0	0	0	94	12	0	0	0
Female medical students (n = 65)	63	1	1	0	0	51	12	1	0	1
Paramedical students (n = 60)	60	0	0	0	0	55	5	0	0	0

Attitudes and practices regarding kidney disease

Attitudes and practices among medical and paramedical students were assessed using a structured questionnaire. Among medical undergraduates, 66.66% believed that they did not have kidney disease, 81.66% believed that eating organic vegetables would reduce the prevalence of kidney disease, and 51.66% believed that water quality was a contributing factor to kidney disease. In terms of treatment preferences, 90% would seek treatment through allopathic medicine, and 92.77% expressed a positive attitude toward organ donation. Among paramedical students, 98.33% believed that they did not have kidney disease, while 100% believed that eating organic vegetables would not influence the prevalence of kidney disease. A majority (96.66%) believed that water quality was a key factor in kidney disease. Regarding treatment, 55% preferred allopathic medicine, while 45% would consider homeopathy. Additionally, 81.66% of paramedical students were supportive of organ donation (Table 2).

**Table 2:** Comparison of Attitudes and Practices Regarding Kidney Disease Among Medical and Paramedical Students Data are represented as N (%). The chi-square test was used for group comparisons. Statistical significance was set at p < 0.05

Attitude/Practice Question	Response	Medical Undergraduate Students (n = 180)	Paramedical Students (n = 60)	Chi-Square Value	P-value
Have you thought that you may have a kidney problem?	Yes	31 (17.2%)	1 (1.7%)	23.88	<0.0001
	Maybe	29 (16.1%)	0 (0%)	23.88	<0.0001
	No	120 (66.7%)	59 (98.3%)	23.88	<0.0001
Do you think eating organically grown vegetables would decrease kidney disease?	Yes	147 (81.7%)	0 (0%)	202.17	<0.0001
	No	0 (0%)	60 (100%)	202.17	<0.0001
Do you think drinking water could be responsible for kidney disease?	Yes	93 (51.7%)	58 (96.7%)	37.15	<0.0001
	Maybe	87 (48.3%)	2 (3.3%)	37.15	<0.0001
If you had kidney disease, would you seek help from allopathy?	Allopathy	162 (90.0%)	33 (55.0%)	33.92	<0.0001
	Homeopathy	18 (10.0%)	27 (45.0%)	33.92	<0.0001
Do you believe in organ donation?	Yes	167 (92.8%)	49 (81.7%)	5.00	0.0253
	No	13 (7.2%)	11 (18.3%)	5.00	0.0253

Symptom patterns and additional findings

The symptom charts revealed that the majority of male, female, and paramedical students reported symptoms only rarely, with few instances of symptoms being reported often or always. Students with abnormal symptom patterns were referred to the medicine outpatient department (OPD) for further evaluation. The mean weight of male medical students was 60 ± 6.9 kg, and female medical students had a mean weight of 54.75 ± 7.9 kg. The mean weight of paramedical students was 50 ± 5 kg. Among male medical students, common symptoms experienced "often" included fatigue (24.4%), dizziness (11.1%), and shortness of breath (10%). Female medical students largely reported minimal symptom burden, with most symptoms marked as "rarely" and only mild increases in shortness of breath (12.3%) and nocturia (3.1%). Paramedical students similarly reported low symptom frequency, though a slightly higher proportion noted difficulty thinking clearly (18.3%), puffy face (16.7%), and feeling sick in the stomach (35%). Notably, classic uremic symptoms such as ammonia-like breath odor, foamy urine, or hematuria were almost universally absent across all groups. No participants reported a personal history of kidney disease. Two students reported a past history of kidney stones, and one student reported a family history of kidney disease.

## Discussion

Asymptomatic persistent proteinuria may be the earliest indicator of underlying kidney disease. When accompanied by hypertension, although blood pressure was recorded during data collection for all participants, the mean values did not indicate hypertension in any individual, and no cases met the diagnostic threshold. Therefore, BP values were not detailed in the Results section to avoid overstatement; it significantly accelerates the progression of renal dysfunction. However, existing literature predominantly focuses on screening individuals over the age of 35, with limited data evaluating the utility of early screening in younger populations. This gap is particularly concerning in developing countries, where public awareness about kidney disease remains low and organized screening programs are often absent. The early identification of urinary abnormalities in asymptomatic individuals, especially among the young who are at future risk of developing CKD, is crucial for timely intervention and prevention [11,12].

In a study by Chhetri and Shah, 5% of female college students had proteinuria [8]. Bakr et al. reported proteinuria in 1.3% of the participants [13], while a Bolivian study by Plata et al. noted urinary abnormalities in 30.3% of their cohort [14]. Other global studies revealed a lower prevalence of asymptomatic urinary abnormalities among schoolchildren, such as 0.62% in Japan, 0.3% in Taiwan, 1.9% in Malaysia, and 5.25% in Nigeria [15-18]. The prevalence appears to increase with age, with studies reporting rates of 2.13% in Egypt, 3.5% in Northern Iran, and 2.6% in India [19-21]. Notably, proteinuria is recognized as one of the strongest predictors of renal function decline and warrants further investigation to detect and manage CKD at an early stage.

Japan stands out as a model for proactive screening, having implemented annual urinalysis for all working adults since 1972 and for all residents over 40 years of age since 1982 [15,22]. Attitudes toward organ donation among healthcare professionals also vary significantly. In a study by Alsaied et al., only 24% of physicians and 20.2% of nurses were willing to donate a kidney to a family member, compared to 44.3% of technicians [23]. While overall support for organ donation stood at 83%, more than half of the respondents still expressed a desire to be buried with all their organs intact, highlighting the cultural and psychological barriers that persist even within the medical community. Anees et al. reported that most doctors demonstrated poor to average knowledge and practice regarding kidney disease [9]. Many also perceived nephrology services in their institutions as inadequate and stressed the need for the inclusion of nephrology education during undergraduate training. They advocated for dedicated nephrology departments to enhance awareness and service delivery.

Strengths and limitations

This study's strength lies in its dual approach, combining knowledge and attitude evaluation with clinical urine screening in a high-prevalence region. It provides preliminary insight into the awareness levels of future healthcare providers. However, key limitations include a single-center design and relatively small sample size, limiting generalizability, and a cross-sectional nature, preventing long-term follow-up. The use of urine dipstick testing without confirmatory measures such as microalbuminuria or serum creatinine and the lack of detailed dietary, exercise, and menstrual history may affect dipstick results. Despite these limitations, the study highlights the value of early, low-cost screening and the need for greater education on kidney health in undergraduate medical and paramedical curricula.

## Conclusions

This study emphasizes the importance of early screening and awareness of kidney disease among asymptomatic young healthcare professionals. Urine dipstick testing revealed proteinuria in a significant number of participants, suggesting early signs of renal dysfunction. In our study, urine dipstick testing was used only as a preliminary screening tool for early urinary abnormalities in asymptomatic individuals, not to diagnose CKD. The participants with abnormal findings were referred for further evaluation and not labelled as having renal dysfunction. While medical students demonstrated fair knowledge, paramedical students showed lower awareness, underscoring the need for improved education. Attitudinal gaps regarding treatment and organ donation were also evident. These findings highlight the necessity of incorporating kidney health education and low-cost screening tools into undergraduate healthcare training. Early identification and awareness can serve as effective strategies in preventing the future burden of chronic kidney disease, especially in high-risk regions such as North Coastal Andhra Pradesh.
